# Crystal structure of magnesium selenate hepta­hydrate, MgSeO_4_·7H_2_O, from neutron time-of-flight data

**DOI:** 10.1107/S1600536814018698

**Published:** 2014-08-23

**Authors:** A. Dominic Fortes, Matthias J. Gutmann

**Affiliations:** a Department of Earth Sciences, University College London, Gower Street, London WC1E 6BT, England; b Department of Earth and Planetary Sciences, Birkbeck, University of London, Malet Street, London WC1E 7HX, England; c ISIS Facility, STFC Rutherford Appleton Laboratory, Harwell Science and Innovation Campus, Chilton, Didcot, Oxfordshire OX11 0QX, England

**Keywords:** crystal structure, magnesium selenate hepta­hydrate, neutron Laue diffraction, hydrogen bonding

## Abstract

MgSeO_4_·7H_2_O is isostructural with the analogous sulfate, MgSO_4_·7H_2_O, consisting of isolated [Mg(H_2_O)_6_]^2+^ octa­hedra and [SeO_4_]^2−^ tetra­hedra, linked by O—H⋯O hydrogen bonds, with a single inter­stitial lattice water mol­ecule. As in the sulfate, the [Mg(H_2_O)_6_]^2+^ coordination octa­hedron is elongated along one axis due to the tetra­hedral coordination of the two apical water mol­ecules; these have Mg—O distances of ∼2.10 Å, whereas the remaining four trigonally coordinated water mol­ecules have Mg—O distances of ∼2.05 Å. The mean Se—O bond length is 1.641 Å and is in excellent agreement with other selenates. The unit-cell volume of MgSeO_4_·7H_2_O at 10 K is 4.1% larger than that of the sulfate at 2 K, although this is not uniform; the greater part of the expansion is along the *a* axis of the crystal.

## Abstract 2

The structure of MgSeO_4_·7H_2_O has been determined by single-crystal Laue diffraction methods using pulsed neutron radiation. The compound is isostructural with the sulfate analogue, MgSO_4_·7H_2_O.

## Chemical context   

Since their discovery almost two hundred years ago, the hepta­hydrates of divalent metal selenates have received scant attention. This is in stark contrast with the *M* ^2+^SeO_4_ hexa­hydrates, which have been extensively characterized, including studies of their morphology and optical properties (Topsøe & Christiansen, 1874 ▶), their crystal structures (Stadnicka *et al.*, 1988 ▶; Kolitsch, 2002 ▶), their formation of isomorphous solution series (*e.g*., Ojkova *et al.*, 1990 ▶: Stoilova *et al.*, 1995 ▶) and their dehydration properties (Nabar & Paralkar, 1975 ▶: Stoilova & Koleva, 1995 ▶). In part this may be due to the fact that the hepta­hydrates must be prepared at lower temperatures. Nevertheless, it is striking that the only information concerning their crystal structures, namely their apparent isomorphism with the *M* ^2+^SO_4_ hepta­hydrates, has remained largely unaltered since the observations made prior to 1830 by Berzelius and his student Mitscherlich, which is that MgSeO_4_·7H_2_O forms deliquescent four-sided prismatic crystals below 288 K (*e.g*., Berzelius, 1818 ▶, 1829 ▶). The only known goniometric data relate to FeSeO_4_·7H_2_O and CoSeO_4_·7H_2_O (Wohlwill, 1860 ▶: Topsøe, 1870 ▶: Tutton, 1918 ▶), which are isomorphous with the monoclinic series of *M* ^2+^SO_4_ hepta­hydrates. It is worth stating that MgMoO_4_·5H_2_O is isomorphous with both the sulfate, chromate and selenate analogues but is not iso*structural* with them [Bars *et al.*, 1977 ▶; see also Lima-de-Faria *et al.* (1990 ▶) for further discussion of these nomenclature], so the occurrence of MgSeO_4_·7H_2_O as acicular rhombic prisms is no guarantee that it is isostructural with the sulfate salt. Additional confusion arises from conflicting observations of the MgSeO_4_–H_2_O binary phase diagram (Meyer & Aulich, 1928 ▶: Klein, 1940 ▶), including our own recent discovery of hitherto unknown hydrates (containing 9H_2_O and 11H_2_O) below 273 K (Fortes, 2014 ▶).

As part of a wider study into low-temperature crystal hydrates of MgSeO_4_ and related compounds (Fortes *et al.*, 2013 ▶) we synthesised the title compound and carried out a single-crystal neutron diffraction experiment in order to determine its structure.

## Structural commentary   

**Figure 1:**
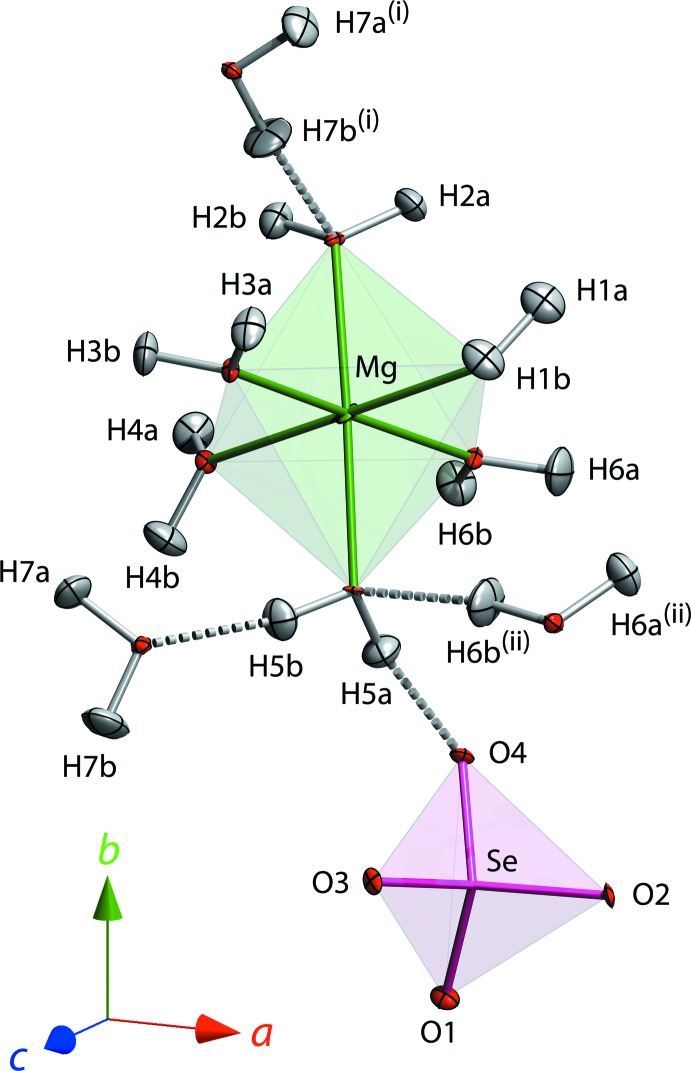
Asymmetric unit of MgSeO_4_·7H_2_O with anisotropic displacement ellipsoids drawn at the 50% probability level (75% for Mg and the selenate O atoms to aid visibility). Dashed rods indicate hydrogen bonds. The superscripts (i) and (ii) denote, respectively, the symmetry operations [1 − *x*, 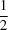 + *y*, 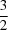 − *z*] and [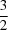 − *x*, 1 − *y*, 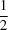 + *z*].

**Figure 2:**
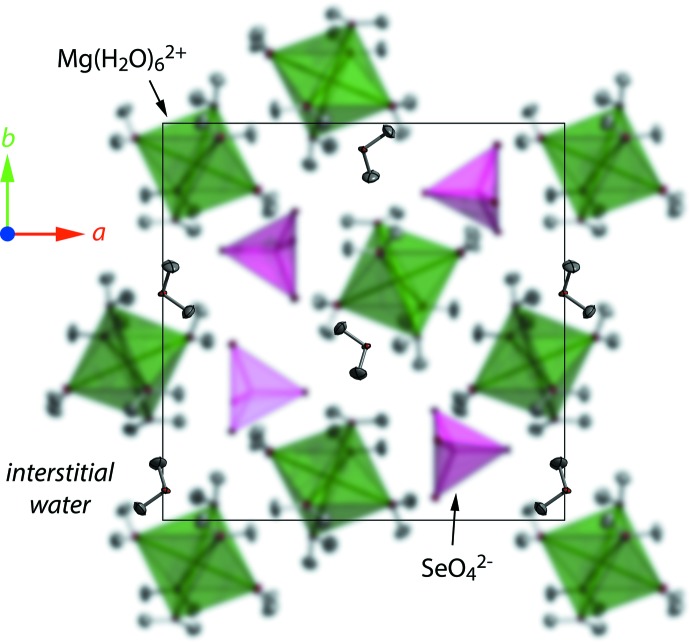
Packing of the polyhedra and inter­stitial water in MgSeO_4_·7H_2_O viewed down the *c*-axis. The polyhedral ions have been blurred in order to emphasize the location of the inter­stitial water mol­ecules.

The crystal structure (Fig. 1 ▶) is isostructural with that of the sulfate, having isolated [Mg(H_2_O)_6_]^2+^ octa­hedra and [SeO_4_]^2−^ tetra­hedra linked by a framework of moderately strong hydrogen bonds (H⋯O from 1.692 to 1.946 Å; Table 1 ▶). The seventh water mol­ecule is coordinated to neither Mg nor Se, occupying a ‘void’ between the polyhedral ions and donating comparatively weak (*i.e.*, long and non-linear) hydrogen bonds (Fig. 2 ▶, Table 1 ▶). The [Mg(H_2_O)_6_]^2+^ octa­hedron is slightly elongated along the O*W*2 – Mg – O*W*5 axis, the respective Mg—O distances being 2.101 Å (average) compared with 2.046 Å (average) for the other four ‘equatorial’ water mol­ecules (Table 2 ▶). This distortion was also noted in the sulfate by Baur (1964 ▶) and is manifested in subsequent neutron single-crystal and powder diffraction studies (Ferraris *et al.*, 1973 ▶: Fortes *et al.*, 2006 ▶). The difference is due to the tetra­hedral coordination of O*W*2 and O*W*5; both of these water mol­ecules (in addition to being Mg-coordin­ated) donate two hydrogen bonds and accept one hydrogen bond, from O*W*7 and O*W*6 respectively. The four ‘equatorial’ water mol­ecules donate but do not accept any hydrogen bonds. In the sulfate at 2 K (Fortes *et al.*, 2006 ▶), the average equatorial Mg—O distances were found to be 2.029 Å and the average axial Mg—O distances to be 2.100 Å (2.056 and 2.102 Å at room temperature; Ferraris *et al.*, 1973 ▶; Calleri *et al.*, 1984 ▶).

The [SeO_4_]^2−^ tetra­hedron exhibits a similar property in that the bond lengths are influenced by the hydrogen-bond coord­ination. Two of the oxygen atoms (O1 and O3) accept two hydrogen bonds and have mean Se—O bond lengths of 1.631 Å, whereas the other two oxygen atoms (O2 and O4) accept three hydrogen bonds and have a mean Se—O bond length of 1.652 Å. This distinction is not readily apparent in any of the data pertaining to the sulfate, but it is worth observing that the neutron scattering cross-section of selenium is almost three times greater than that of sulfur so our result should be considered more accurate. The mean Se—O bond length of 1.641 Å is in excellent agreement with other similar high-precision analyses of selenate crystals (Kolitsch, 2001 ▶, 2002 ▶; Weil & Bonneau, 2014 ▶).

Overall, the unit-cell volume of the selenate at 10 K is 4.1% larger than the sulfate analogue (deuterated) at 2 K. This expansion is not isotropic, however, with the greatest proportion being along the *a* axis of the crystal. We find that the *a* axis is 2.7% longer, the *b* axis 1.0% longer, and the *c* axis 0.3% longer in the selenate than the sulfate. It is not readily apparent from examination of the structure why this should be so. The magnitude of the volumetric strain is virtually identical to that found in MgSeO_4_·11H_2_O (4.1% larger than the sulfate analogue; Fortes, 2014 ▶) and somewhat less than is observed in, for example, CuSeO_4_·5H_2_O (5.1% larger than the equivalent sulfate; Kolitsch, 2001 ▶) or MgSeO_4_·6H_2_O (5.2%; Kolitsch, 2002 ▶).

## Synthesis and crystallization   

In our initial attempts to make MgSeO_4_ we employed the widely cited method of reacting basic Mg-carbonate with aqueous selenic acid (*e.g.*, Stoilova & Koleva, 1995 ▶), but this was found to leave a substantial amount of acid in solution, giving a pink-coloured viscous liquid with a sour odour, which yielded an intimate mixture of MgSeO_4_·6H_2_O and Mg(HSeO_3_)_2_·4H_2_O crystals (*cf*., Kolitsch, 2002 ▶; Mička *et al.*, 1996 ▶) even after repeated re-crystallization and treatment with aqueous H_2_O_2_. Consequently, we prepared an aqueous solution of magnesium selenate by stirring MgO into a solution of H_2_SeO_4_ (Sigma–Aldrich 481513, 40%_wt_ diluted further in its own weight of distilled water) heated to 340 K. This reaction is much less dramatic than is the case when Mg-carbonate is used and the only clear indication that it has run to completion is the pH of the solution, which changed from 0.11 to 8.80. After a period of evaporation in the open air, the solution precipitates cm-sized crystals of MgSeO_4_·6H_2_O. After a further round of recrystallization from distilled water the phase purity of the hexa­hydrate was verified both by X-ray powder diffraction and Raman spectroscopy.

Finally, crystalline MgSeO_4_·6H_2_O was dissolved in distilled water to a concentration of 35%_wt_ MgSeO_4_ at 333 K, and this liquid was left to evaporate in a refrigerated workshop at 269 K. After two days, slender prismatic crystals indistinguishable in habit from MgSO_4_·7H_2_O, appeared. One of these was removed from the liquid, dried on filter paper and cut into a pair of fragments each with dimensions 1 x 1 x 4 mm. The two fragments were placed side-by-side in an aluminium foil pouch suspended inside a standard thin-walled vanadium sample can (6 mm inner diameter). The lid of the can was sealed with indium wire and was then transported to the ISIS neutron source immersed in liquid nitro­gen.

The sample can was screwed onto a standard centre stick and inserted into a pre-cooled Closed-Cycle Refrigerator (CCR) already mounted on the SXD beam-line (Keen *et al.*, 2006 ▶). Initial data collection as the sample was cooled from 200 K down to 10 K revealed strong reflections from both crystals that could be indexed with an ortho­rhom­bic unit cell of similar shape but roughly 4% larger than that of MgSO_4_·7H_2_O. After cooling to 10 K data were collected with the crystals in four discrete orientations with respect to the incident beam, optimizing the coverage of reciprocal space, with integration times of 1600 µAhr each (roughly 10 h per frame at typical ISIS beam intensity). The peaks were indexed and integrated using the instrument software, *SXD2001* (Gutmann, 2005 ▶) and exported in a format suitable for analysis using *SHELX2014* (Sheldrick, 2008 ▶; Gruene *et al.*, 2014 ▶).

Upon completion of the experiment, crystals of the title compound that had been stored in a glass vial at 253 K for ten days were analysed by means of X-ray powder diffraction. This measurement, carried out on a custom Peltier cold stage (Wood *et al.*, 2012 ▶) at 253 K, revealed that the hepta­hydrate had transformed completely to the newly reported MgSeO_4_·9H_2_O (Fortes, 2014 ▶), thus providing some initial insight into the relative stability of the two compounds.

## Refinement   

Crystal data, data collection and structure refinement details are summarized in Table 3 ▶. Structure refinement with *SHELXL* using the model obtained at 2 K for the deuterated MgSO_4_ analogue (Fortes *et al.*, 2006 ▶) based on earlier work (Baur, 1964 ▶: Ferraris *et al.*, 1973 ▶: Calleri *et al.*, 1984 ▶) yielded a good fit with no density residuals larger than 4.5% of the nuclear scattering density due to a hydrogen atom. No restraints were used and all anisotropic temperature factors were refined independently.

## Supplementary Material

Crystal structure: contains datablock(s) I, New_Global_Publ_Block. DOI: 10.1107/S1600536814018698/wm5046sup1.cif

Structure factors: contains datablock(s) I. DOI: 10.1107/S1600536814018698/wm5046Isup2.hkl

CCDC reference: 1019812

Additional supporting information:  crystallographic information; 3D view; checkCIF report

## Figures and Tables

**Table 1:** Hydrogen-bond geometry (Å, °)

*D*—H⋯*A*	*D*—H	H⋯*A*	*D*⋯*A*	*D*—H⋯*A*
O*W*1—H1*A*⋯O3^i^	0.969 (16)	1.692 (16)	2.659 (9)	175.4 (13)
O*W*1—H1*B*⋯O4^ii^	0.968 (16)	1.757 (15)	2.724 (9)	175.1 (11)
O*W*2—H2*A*⋯O2^i^	0.983 (14)	1.781 (15)	2.757 (9)	171.0 (11)
O*W*2—H2*B*⋯O4^iii^	0.984 (11)	1.753 (11)	2.732 (7)	172.4 (10)
O*W*3—H3*A*⋯O2^ii^	0.976 (13)	1.889 (13)	2.861 (8)	174.5 (12)
O*W*3—H3*B*⋯O3^iv^	0.985 (9)	1.708 (9)	2.692 (6)	177.4 (14)
O*W*4—H4*A*⋯O1^iii^	0.976 (14)	1.720 (15)	2.688 (9)	170.9 (11)
O*W*4—H4*B*⋯O2^v^	0.964 (13)	1.927 (11)	2.861 (7)	162.3 (15)
O*W*5—H5*A*⋯O4	0.976 (14)	1.874 (14)	2.839 (8)	169.6 (10)
O*W*5—H5*B*⋯O*W*7	0.967 (15)	1.786 (14)	2.742 (9)	169.4 (10)
O*W*6—H6*A*⋯O*W*5^i^	0.976 (10)	1.875 (10)	2.841 (6)	170.3 (12)
O*W*6—H6*B*⋯O*W*7^vi^	0.982 (14)	1.809 (13)	2.787 (8)	173.4 (11)
O*W*7—H7*A*⋯O1^iv^	0.973 (10)	1.858 (12)	2.790 (7)	159.5 (14)
O*W*7—H7*B*⋯O*W*2^vii^	0.955 (13)	1.946 (16)	2.866 (8)	161.2 (15)

Symmetry codes: (i) 
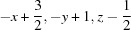
; (ii) 
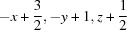
; (iii) 
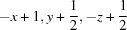
; (iv) 
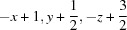
; (v) 
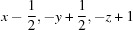
; (vi) 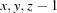; (vii) 
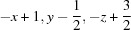
.

**Table 2:** Selected bond lengths (Å)

Se1—O1	1.630 (6)	Mg1—O*W*1	2.045 (6)
Se1—O3	1.631 (8)	Mg1—O*W*3	2.046 (10)
Se1—O2	1.642 (4)	Mg1—O*W*6	2.058 (9)
Se1—O4	1.661 (7)	Mg1—O*W*5	2.097 (8)
Mg1—O*W*4	2.037 (6)	Mg1—O*W*2	2.104 (8)

**Table 3:** Experimental details

Crystal data	
Chemical formula	[Mg(H_2_O)_6_](SeO_4_)(H_2_O)
*M*_r_	293.38
Crystal system, space group	Orthorhombic, *P*2_1_2_1_2_1_
Temperature (K)	10
*a*, *b*, *c* (Å)	12.234 (4), 12.020 (4), 6.809 (3)
*V* (Å^3^)	1001.3 (6)
*Z*	4
Radiation type	Neutron, λ = 0.48–7.0 Å
μ (mm^−1^)	0.48 + 0.0036 * λ
Crystal size (mm)	1.00 × 1.00 × 4.00

Data collection	
Diffractometer	SXD diffractometer
Absorption correction	Numerical. The linear absorption coefficient is wavelength dependent and is calculated as: μ = 0.4823 + 0.0036 * λ [mm^−1^] as determined by Gaussian integration in *SXD2001* (Gutmann, 2005 ▶)
No. of measured, independent and observed [*I* > 2σ(*I*)] reflections	4337, 4337, 4337

Refinement	
*R*[*F* ^2^ > 2σ(*F* ^2^)], *wR*(*F* ^2^), *S*	0.072, 0.197, 1.08
No. of reflections	4337
No. of parameters	252
H-atom treatment	All H-atom parameters refined
	*w* = 1/[σ^2^(*F*_o_^2^) + (0.1399*P*)^2^ + 21.2928*P*] where *P* = (*F*_o_^2^ + 2*F*_c_^2^)/3
Δρ_max_, Δρ_min_ (fermi Å^−3^)	2.06, −1.72
Absolute structure	All *f*′′ are zero, so absolute structure could not be determined

Computer programs: *SXD2001* (Gutmann, 2005 ▶), *SHELXS2014* and *SHELXL2014* (Gruene *et al.*, 2014 ▶), *DIAMOND* (Putz & Brandenburg, 2006 ▶) and *publCIF* (Westrip, 2010 ▶).

## supplementary crystallographic information

### Crystal data

**Table:**

[Mg(H_2_O)_6_](SeO_4_)(H_2_O)	*F*(000) = 592
*M**_r_* = 293.38	*D*_x_ = 1.946 Mg m^−^^3^
Orthorhombic, *P*2_1_2_1_2_1_	Neutron radiation, λ = 0.48–7.0 Å
*a* = 12.234 (4) Å	Cell parameters from 550 reflections
*b* = 12.020 (4) Å	µ = 0.48 + 0.0036 * λ mm^−^^1^
*c* = 6.809 (3) Å	*T* = 10 K
*V* = 1001.3 (6) Å^3^	Rhomboid, colourless
*Z* = 4	4.00 × 1.00 × 1.00 mm

### Data collection

**Table:**

SXD diffractometer	4337 independent reflections
Radiation source: ISIS neutron spallation source	4337 reflections with *I* > 2σ(*I*)
time–of–flight LAUE diffraction scans	θ_max_ = 84.5°, θ_min_ = 0.001°
Absorption correction: numerical The linear absorption coefficient is wavelength dependent and is calculated as: µ = 0.4823 + 0.0036 * λ [mm^-1^] as determined by Gaussian integration in *SXD2001* (Gutmann, 2005)	*h* = −32→30
*T*_min_ = ?, *T*_max_ = ?	*k* = −31→20
4337 measured reflections	*l* = −11→6

### Refinement

**Table:**

Refinement on *F*^2^	Hydrogen site location: difference Fourier map
Least-squares matrix: full	All H-atom parameters refined
*R*[*F*^2^ > 2σ(*F*^2^)] = 0.072	*w* = 1/[σ^2^(*F*_o_^2^) + (0.1399*P*)^2^ + 21.2928*P*] where *P* = (*F*_o_^2^ + 2*F*_c_^2^)/3
*wR*(*F*^2^) = 0.197	(Δ/σ)_max_ < 0.001
*S* = 1.08	Δρ_max_ = 2.06 e Å^−^^3^
4337 reflections	Δρ_min_ = −1.72 e Å^−^^3^
252 parameters	Extinction correction: *SHELXL2014* (Gruene et al., 2014), Fc^*^=kFc[1+0.001xFc^2^λ^3^/sin(2θ)]^-1/4^
0 restraints	Extinction coefficient: 0.0026 (3)
Primary atom site location: structure-invariant direct methods	Absolute structure: All f" are zero, so absolute structure could not be determined

### Special details

**Table:**

Experimental. For peak integration a local UB matrix refined for each frame, using approximately 50 reflections from each of the 11 detectors. Hence _cell_measurement_reflns_used 550 For final cell dimensions a weighted average of all local cells was calculated Because of the nature of the experiment, it is not possible to give values of theta_min and theta_max for the cell determination. The same applies for the wavelength used for the experiment. The range of wavelengths used was 0.48–7.0 Angstroms, BUT the bulk of the diffraction information is obtained from wavelengths in the range 0.7–2.5 Angstroms. The data collection procedures on the SXD instrument used for the single-crystal neutron data collection are most recently summarized in the Appendix to the following paper Wilson, C. C. (1997). J. Mol. Struct. 405, 207–217
Geometry. All e.s.d.s (except the e.s.d. in the dihedral angle between two l.s. planes) are estimated using the full covariance matrix. The cell e.s.d.s are taken into account individually in the estimation of e.s.d.s in distances, angles and torsion angles; correlations between e.s.d.s in cell parameters are only used when they are defined by crystal symmetry. An approximate (isotropic) treatment of cell e.s.d.s is used for estimating e.s.d.s involving l.s. planes.
Refinement. The variable wavelength nature of the data collection procedure means that sensible values of _diffrn_reflns_theta_min & _diffrn_reflns_theta_max cannot be given instead the following limits are given _diffrn_reflns_sin(theta)/lambda_min 0.06 _diffrn_reflns_sin(theta)/lambda_max 1.38 _refine_diff_density_max/min is given in Fermi per angstrom cubed not electons per angstrom cubed. Another way to consider the _refine_diff_density_ is as a percentage of the scattering density of a given atom: _refine_diff_density_max = 4.5% of hydrogen _refine_diff_density_min = -3.8% of hydrogen Refinement of *F*^2^ against ALL reflections. The weighted *R*-factor *wR* and goodness of fit *S* are based on *F*^2^, conventional *R*-factors *R* are based on *F*, with *F* set to zero for negative *F*^2^. The threshold expression of *F*^2^ > σ(*F*^2^) is used only for calculating *R*-factors(gt) *etc*. and is not relevant to the choice of reflections for refinement. *R*-factors based on *F*^2^ are statistically about twice as large as those based on *F*, and *R*- factors based on ALL data will be even larger. For comparison, the calculated *R*-factor based on *F* is 0.0578 for the 1606 unique reflections obtained after merging to generate the Fourier map.

### Fractional atomic coordinates and isotropic or equivalent isotropic displacement parameters (Å^2^)

**Table:**

	*x*	*y*	*z*	*U*_iso_*/*U*_eq_	
Se1	0.72001 (19)	0.1764 (3)	0.4966 (7)	0.0005 (6)	
O1	0.6727 (3)	0.0565 (4)	0.4240 (10)	0.0045 (10)	
O2	0.8540 (3)	0.1781 (4)	0.4840 (10)	0.0039 (10)	
O3	0.6788 (3)	0.2016 (4)	0.7201 (10)	0.0032 (9)	
O4	0.6714 (3)	0.2769 (4)	0.3540 (10)	0.0037 (10)	
Mg1	0.5841 (3)	0.6050 (4)	0.4601 (11)	0.0028 (11)	
OW1	0.7361 (3)	0.6719 (5)	0.5016 (11)	0.0053 (10)	
OW2	0.5312 (3)	0.7470 (4)	0.3064 (10)	0.0046 (10)	
OW3	0.5370 (3)	0.6718 (5)	0.7231 (10)	0.0052 (10)	
OW4	0.4319 (3)	0.5399 (4)	0.4219 (10)	0.0049 (11)	
OW5	0.6304 (3)	0.4595 (4)	0.6082 (10)	0.0042 (10)	
OW6	0.6434 (3)	0.5384 (4)	0.2028 (11)	0.0059 (11)	
OW7	0.5042 (3)	0.4328 (4)	0.9377 (11)	0.0048 (10)	
H1A	0.7663 (8)	0.7214 (10)	0.403 (2)	0.019 (2)	
H1B	0.7660 (8)	0.6934 (9)	0.628 (2)	0.017 (3)	
H2A	0.5789 (8)	0.7721 (9)	0.199 (2)	0.017 (2)	
H2B	0.4571 (7)	0.7511 (9)	0.251 (2)	0.016 (2)	
H3A	0.5772 (7)	0.7242 (9)	0.805 (2)	0.018 (3)	
H3B	0.4584 (7)	0.6826 (10)	0.748 (2)	0.018 (2)	
H4A	0.3879 (7)	0.5493 (9)	0.304 (2)	0.017 (2)	
H4B	0.4068 (8)	0.4717 (9)	0.481 (2)	0.020 (3)	
H5A	0.6362 (7)	0.3931 (9)	0.526 (2)	0.019 (3)	
H5B	0.5863 (8)	0.4406 (9)	0.721 (2)	0.018 (2)	
H6A	0.7205 (7)	0.5302 (10)	0.170 (3)	0.026 (3)	
H6B	0.5992 (8)	0.4994 (10)	0.104 (2)	0.019 (3)	
H7A	0.4395 (7)	0.4789 (9)	0.953 (2)	0.021 (3)	
H7B	0.4843 (8)	0.3642 (9)	0.998 (3)	0.023 (3)	

### Atomic displacement parameters (Å^2^)

**Table:**

	*U*^11^	*U*^22^	*U*^33^	*U*^12^	*U*^13^	*U*^23^
Se1	0.0004 (7)	0.0005 (10)	0.0006 (18)	−0.0001 (7)	−0.0003 (10)	−0.0002 (15)
O1	0.0057 (12)	0.0042 (19)	0.004 (3)	−0.0009 (12)	−0.0004 (16)	−0.0005 (19)
O2	0.0013 (10)	0.0051 (18)	0.005 (3)	−0.0001 (11)	0.0015 (14)	0.002 (2)
O3	0.0041 (13)	0.0042 (19)	0.001 (3)	−0.0006 (11)	0.0013 (15)	−0.0011 (19)
O4	0.0046 (13)	0.0025 (18)	0.004 (3)	0.0004 (12)	0.0005 (15)	0.0016 (19)
Mg1	0.0027 (12)	0.0021 (17)	0.003 (3)	0.0008 (11)	−0.0005 (14)	−0.0019 (19)
OW1	0.0046 (12)	0.008 (2)	0.003 (3)	−0.0021 (12)	0.0007 (17)	−0.001 (3)
OW2	0.0038 (12)	0.0028 (19)	0.007 (3)	0.0004 (11)	−0.0016 (16)	0.000 (2)
OW3	0.0038 (11)	0.007 (2)	0.005 (3)	−0.0004 (13)	0.0005 (15)	−0.001 (2)
OW4	0.0042 (13)	0.006 (2)	0.005 (3)	−0.0011 (13)	−0.0017 (14)	0.000 (2)
OW5	0.0057 (13)	0.0010 (19)	0.006 (3)	0.0000 (12)	0.0002 (14)	0.001 (2)
OW6	0.0046 (13)	0.006 (2)	0.007 (3)	−0.0001 (13)	0.0006 (16)	−0.002 (2)
OW7	0.0064 (14)	0.0040 (19)	0.004 (3)	0.0009 (12)	0.0009 (15)	0.000 (2)
H1A	0.022 (4)	0.020 (5)	0.016 (7)	−0.005 (3)	−0.004 (4)	−0.002 (5)
H1B	0.020 (4)	0.019 (5)	0.013 (8)	−0.006 (3)	−0.004 (4)	−0.002 (5)
H2A	0.019 (4)	0.014 (4)	0.018 (7)	−0.002 (3)	0.007 (4)	0.004 (5)
H2B	0.013 (3)	0.017 (4)	0.018 (7)	0.001 (3)	0.000 (3)	−0.003 (5)
H3A	0.015 (3)	0.019 (5)	0.019 (7)	−0.003 (3)	0.001 (4)	−0.012 (5)
H3B	0.009 (3)	0.024 (5)	0.021 (7)	0.001 (3)	0.005 (3)	−0.002 (5)
H4A	0.016 (3)	0.021 (5)	0.013 (7)	0.001 (3)	−0.006 (3)	−0.002 (5)
H4B	0.022 (4)	0.016 (4)	0.021 (8)	−0.007 (3)	−0.001 (4)	0.005 (5)
H5A	0.021 (3)	0.014 (4)	0.021 (8)	0.002 (3)	0.003 (4)	−0.004 (5)
H5B	0.020 (4)	0.022 (5)	0.011 (7)	−0.001 (3)	0.002 (4)	0.001 (5)
H6A	0.009 (3)	0.030 (6)	0.039 (10)	0.002 (3)	0.004 (4)	0.001 (6)
H6B	0.018 (3)	0.023 (5)	0.015 (8)	−0.005 (3)	−0.002 (4)	−0.004 (5)
H7A	0.015 (3)	0.018 (5)	0.030 (9)	0.007 (3)	0.001 (4)	0.007 (5)
H7B	0.026 (4)	0.016 (5)	0.028 (9)	−0.003 (3)	−0.002 (5)	0.013 (6)

### Geometric parameters (Å, º)

**Table:**

Se1—O1	1.630 (6)	OW2—H2A	0.983 (14)
Se1—O3	1.631 (8)	OW2—H2B	0.984 (11)
Se1—O2	1.642 (4)	OW3—H3A	0.976 (13)
Se1—O4	1.661 (7)	OW3—H3B	0.985 (9)
Mg1—OW4	2.037 (6)	OW4—H4B	0.964 (13)
Mg1—OW1	2.045 (6)	OW4—H4A	0.976 (14)
Mg1—OW3	2.046 (10)	OW5—H5B	0.967 (15)
Mg1—OW6	2.058 (9)	OW5—H5A	0.976 (14)
Mg1—OW5	2.097 (8)	OW6—H6A	0.976 (10)
Mg1—OW2	2.104 (8)	OW6—H6B	0.982 (14)
OW1—H1B	0.968 (16)	OW7—H7B	0.955 (13)
OW1—H1A	0.969 (16)	OW7—H7A	0.973 (10)
			
O1—Se1—O3	109.7 (3)	OW5—Mg1—OW2	177.3 (3)
O1—Se1—O2	110.5 (3)	H1B—OW1—H1A	107.8 (12)
O3—Se1—O2	110.8 (4)	H1B—OW1—Mg1	124.8 (9)
O1—Se1—O4	109.8 (4)	H1A—OW1—Mg1	119.5 (9)
O3—Se1—O4	107.4 (3)	H2A—OW2—H2B	104.2 (12)
O2—Se1—O4	108.6 (3)	H2A—OW2—Mg1	115.9 (7)
OW4—Mg1—OW1	179.2 (5)	H2B—OW2—Mg1	121.0 (7)
OW4—Mg1—OW3	90.3 (3)	H3A—OW3—H3B	108.0 (11)
OW1—Mg1—OW3	88.9 (4)	H3A—OW3—Mg1	127.8 (8)
OW4—Mg1—OW6	93.7 (3)	H3B—OW3—Mg1	118.5 (10)
OW1—Mg1—OW6	87.2 (3)	H4B—OW4—H4A	105.5 (11)
OW3—Mg1—OW6	175.7 (3)	H4B—OW4—Mg1	124.4 (8)
OW4—Mg1—OW5	89.3 (3)	H4A—OW4—Mg1	124.5 (8)
OW1—Mg1—OW5	90.9 (3)	H5B—OW5—H5A	107.7 (11)
OW3—Mg1—OW5	89.0 (4)	H5B—OW5—Mg1	115.3 (7)
OW6—Mg1—OW5	89.4 (3)	H5A—OW5—Mg1	115.3 (10)
OW4—Mg1—OW2	88.1 (3)	H6A—OW6—H6B	109.0 (13)
OW1—Mg1—OW2	91.7 (3)	H6A—OW6—Mg1	125.2 (11)
OW3—Mg1—OW2	91.7 (3)	H6B—OW6—Mg1	125.1 (8)
OW6—Mg1—OW2	90.0 (4)	H7B—OW7—H7A	103.7 (11)

### Hydrogen-bond geometry (Å, º)

**Table:**

*D*—H···*A*	*D*—H	H···*A*	*D*···*A*	*D*—H···*A*
O*W*1—H1*A*···O3^i^	0.969 (16)	1.692 (16)	2.659 (9)	175.4 (13)
O*W*1—H1*B*···O4^ii^	0.968 (16)	1.757 (15)	2.724 (9)	175.1 (11)
O*W*2—H2*A*···O2^i^	0.983 (14)	1.781 (15)	2.757 (9)	171.0 (11)
O*W*2—H2*B*···O4^iii^	0.984 (11)	1.753 (11)	2.732 (7)	172.4 (10)
O*W*3—H3*A*···O2^ii^	0.976 (13)	1.889 (13)	2.861 (8)	174.5 (12)
O*W*3—H3*B*···O3^iv^	0.985 (9)	1.708 (9)	2.692 (6)	177.4 (14)
O*W*4—H4*A*···O1^iii^	0.976 (14)	1.720 (15)	2.688 (9)	170.9 (11)
O*W*4—H4*B*···O2^v^	0.964 (13)	1.927 (11)	2.861 (7)	162.3 (15)
O*W*5—H5*A*···O4	0.976 (14)	1.874 (14)	2.839 (8)	169.6 (10)
O*W*5—H5*B*···O*W*7	0.967 (15)	1.786 (14)	2.742 (9)	169.4 (10)
O*W*6—H6*A*···O*W*5^i^	0.976 (10)	1.875 (10)	2.841 (6)	170.3 (12)
O*W*6—H6*B*···O*W*7^vi^	0.982 (14)	1.809 (13)	2.787 (8)	173.4 (11)
O*W*7—H7*A*···O1^iv^	0.973 (10)	1.858 (12)	2.790 (7)	159.5 (14)
O*W*7—H7*B*···O*W*2^vii^	0.955 (13)	1.946 (16)	2.866 (8)	161.2 (15)

Symmetry codes: (i) −*x*+3/2, −*y*+1, *z*−1/2; (ii) −*x*+3/2, −*y*+1, *z*+1/2; (iii) −*x*+1, *y*+1/2, −*z*+1/2; (iv) −*x*+1, *y*+1/2, −*z*+3/2; (v) *x*−1/2, −*y*+1/2, −*z*+1; (vi) *x*, *y*, *z*−1; (vii) −*x*+1, *y*−1/2, −*z*+3/2.

## References

[bb1] Bars, O., Le Marouille, J.-Y. & Grandjean, D. (1977). *Acta Cryst.* B**33**, 1155–1157.

[bb2] Baur, W. H. (1964). *Acta Cryst.* **17**, 1361–1369.

[bb3] Berzelius, J. (1818). *J. Chem. Phys.* **23**, 430–484.

[bb4] Berzelius, J. (1829). *Jahres-Bericht über die Fortschritte der Physichen Wissenschaften* Tübingen.

[bb5] Calleri, M., Gavetti, A., Ivaldi, G. & Rubbo, M. (1984). *Acta Cryst.* B**40**, 218–222.

[bb6] Ferraris, G., Jones, D. W. & Yerkess, J. (1973). *J. Chem. Soc. Dalton Trans.* **8**, 816–821.

[bb7] Fortes, A. D. (2014). *Powder Diffr.* Submitted.

[bb8] Fortes, A. D., Wood, I. G., Alfredsson, M., Vočadlo, L. & Knight, K. S. (2006). *Eur. J. Min.* **18**, 449–462.

[bb9] Fortes, A. D., Wood, I. G. & Gutmann, M. J. (2013). *Acta Cryst.* C**69**, 324–329. 10.1107/S0108270113005751 23579697

[bb10] Gruene, T., Hahn, H. W., Luebben, A. V., Meilleur, F. & Sheldrick, G. M. (2014). *J. Appl. Cryst.* **47**, 462–466. 10.1107/S1600576713027659 PMC3937812 24587788

[bb11] Gutmann, M. J. (2005). *SXD2001* ISIS Facility, Rutherford Appleton Laboratory, Oxfordshire, England.

[bb12] Keen, D. A., Gutmann, M. J. & Wilson, C. C. (2006). *J. Appl. Cryst.* **39**, 714–722.

[bb13] Klein, A. (1940). *Ann. Chim.* **14**, 263–317.

[bb14] Kolitsch, U. (2001). *Acta Cryst.* E**57**, i104–i105.

[bb15] Kolitsch, U. (2002). *Acta Cryst.* E**58**, i3–i5.

[bb16] Lima-de-Faria, J., Hellner, E., Liebau, F., Makovicky, E. & Parthé, E. (1990). *Acta Cryst.* A**46**, 1–11.

[bb17] Meyer, J. & Aulich, W. (1928). *Z. Anorg. Allg. Chem.* **172**, 321–343.

[bb18] Mička, Z., Němec, I. & Vojtíšek, P. (1996). *J. Solid State Chem.* **122**, 338–342.

[bb19] Nabar, M. A. & Paralkar, S. V. (1975). *Thermochim. Acta*, **13**, 93–95.

[bb20] Ojkova, T., Balarew, C. & Staneva, D. (1990). *Z. Anorg. Allg. Chem.* **584**, 217–224.

[bb21] Putz, H. & Brandenburg, K. (2006). *DIAMOND* Crystal Impact GbR, Bonn, Germany.

[bb22] Sheldrick, G. M. (2008). *Acta Cryst.* A**64**, 112–122. 10.1107/S0108767307043930 18156677

[bb23] Stadnicka, K., Glazer, A. M. & Koralewski, M. (1988). *Acta Cryst.* B**44**, 356–361.

[bb24] Stoilova, D. & Koleva, V. (1995). *Thermochim. Acta*, **255**, 33–38.

[bb25] Stoilova, D., Ojkova, T. & Staneva, D. (1995). *Cryst. Res. Technol.* **30**, 3–7.

[bb26] Topsøe, H. (1870). *Krystallografisk-kemiske Undersøgelser over de selensure salte* Dissertation, København, Denmark.

[bb27] Topsøe, H. & Christiansen, C. (1874). *Ann. Chim. Phys. 5e Série*, **1**, 5–99.

[bb28] Tutton, A. E. H. (1918). *Proc. Roy. Soc. London. A*, **94**, 352–361.

[bb29] Weil, M. & Bonneau, B. (2014). *Acta Cryst.* E**70**, 54–57. 10.1107/S1600536814011799 PMC4158548 25249853

[bb30] Westrip, S. P. (2010). *J. Appl. Cryst.* **43**, 920–925.

[bb31] Wohlwill, E. (1860). *Über isomorphe Mischungen der selensauren Salze.* Dissertation, Georg-August Universität Göttingen, Germany.

[bb32] Wood, I. G., Hughes, N. J., Browning, F. & Fortes, A. D. (2012). *J. Appl. Cryst.* **45**, 608–610.

